# Early kinetics of calprotectin in plasma following inguinal hernia surgery

**DOI:** 10.1177/17534259211069635

**Published:** 2022-02-01

**Authors:** Kristina Sejersen, Aleksandra Havelka, Pearl Sanchez Salas, Anders Larsson

**Affiliations:** 1 Department of Medical Sciences, 8097 Uppsala University, Uppsala, Sweden; 2 Unilabs AB, Stockholm, Sweden; 3 Department of Molecular Medicine and Surgery, 27106 Karolinska Institute, Stockholm, Sweden; 4 Gentian Diagnostics AB, Stockholm, Sweden; 5 Department of Surgery, 59585 Gävle Hospital, Region Gävleborg, Sweden

**Keywords:** Calprotectin, kinetics, inflammation, inguinal hernia surgery

## Abstract

Calprotectin is one of the most abundant proteins of neutrophil granulocytes. It is released upon neutrophil activation and is considered a sensitive and clinically useful marker for neutrophil-mediated inflammation, including bacterial infections. However, early kinetics of calprotectin activation following inflammatory activation has hitherto been unknown. The aim of the present study was to determine the early phase of the kinetics of calprotectin, in comparison with the inflammatory markers CRP, IL-6, TNF-α, and procalcitonin, in plasma following a standardized temporary mild inflammatory response, using uncomplicated inguinal hernia surgery as a model. The study cohort consisted of 17 adult patients (15 male and 2 female) undergoing elective surgery for hernia. Values of calprotectin increased significantly at 2 h following surgery, and continued to increase to reach the highest level at 24–36 h after surgery, values still not exceeding upper normal reference level. This contrasts to IL-6 and CRP, for which an elevation was found first later, 4 h and 24–36 h post-surgery, respectively, for IL-6, and CRP. No significant increase was seen for TNF-α, or procalcitonin. The data demonstrate a very rapid and significant but modest increase in calprotectin following induction of mild inflammation, supporting that calprotectin can be useful for early detection of inflammatory response.

## Introduction

Infectious diseases are common and constitute a major global cause of death.^1^ Early diagnosis is clinically important to reduce delay from symptom onset to initiation of adequate medical therapy to reduce risks of protracted infection, sepsis, and their late sequelae,^2^ also to avoid improper use of antibiotics.

In clinical practice, early diagnosis of a bacterial infection which require antibiotic treatment is most frequently based on observation of clinical symptoms, supplemented with leukocyte counts and/or C-reactive protein (CRP) measurement in blood. However, these clinical diagnostic measures result in missing bacterial infections in a considerable proportion approximately 40% of patients.^3^ The neutrophil protein calprotectin has been suggested to serve as a biomarker of potential use to detect bacterial infections at an early stage and was recently reported to distinguish bacterial respiratory infections from those caused by virus or mycoplasma.^4^ Neutrophils are among the first cells to invade inflammatory sites, and contribute to pathogen killing through mechanisms including production of reactive oxygen species (ROS) by the NADPH oxidase, and release of cytotoxic products during the degranulation process and formation of neutrophil extracellular traps. Neutrophils can secrete a considerable number of substances important to attract other immune cells and modulate their effect.^5^ Activation of neutrophils has for long been attributed solely to the presence of pathogens, but later studies demonstrated that it can also be induced by the endogenous factors damage associated molecular pattern (DAMPs) or alarmins.^6^ DAMPs are released by activated phagocytes such as neutrophils and monocytes.^7,8^ Calprotectin, a member of the DAMPs, is abundantly expressed in the cytosol, constituting around 45% of total proteins of circulating neutrophils, and participates in both intra- and extracellular activities.^9^

Calprotectin is known to regulate intracellularly NADPH oxidase activity,^10,11^ the major source of ROS in neutrophils. High concentrations of Calprotectin extracellularly are found at local sites of inflammation or in the serum of patients with inflammatory diseases (e.g. rheumatoid arthritis (RA), cystic fibrosis, or inflammatory bowel disease (IBD))^8,12,13^ and represents a sensitive inflammation biomarker strongly associated with disease level.^14^ Furthermore, studies have demonstrated calprotectin to be a promising biomarker for sepsis.^15–17^ However, the early kinetics of calprotectin in blood/plasma in clinical situations including inflammatory/neutrophil activation has hitherto been unknown. To this end the present study was undertaken to reveal the early kinetics of calprotectin in plasma following elective surgery for uncomplicated hernia as a clinical model for inflammatory response,^18^ including neutrophil activation,^19^ and to compare this kinetics with that for the inflammatory markers CRP, IL-6, TNF-α, and procalcitonin.

## Materials and methods

### Patients

Twenty patients were initially included in the study. As inclusion criteria, adults undergoing elective surgery for uncomplicated hernia with laparoscopic or open surgery, and without signs of inflammatory disease (CRP < 5 mg/l) at entry of study were eligible for participation. For laparoscopic surgery was used transabdominal preperitoneal mesh placement (TAPP) technique or totally extraperitoneal (TEP) approach. The open inguinal surgery was performed using the Lichtenstein technique. For inclusion in the study, patients should give blood samples just before (< 0.5 h) surgery (basal level) and at least one time post-surgery. Exclusion criteria were CRP ≥ 5 mg/l, white blood cell (WBC) count >9.0 × 10^9^/l (≥18 years), known active RA, IBD or other active inflammatory disease known at time for inclusion. In addition, children (age < 18 yr) and patients who could not give informed consent were excluded from the study. The patients were admitted to Department of Surgery, Gävle Hospital, Region Gävleborg, Sweden. Blood samples were collected in BD Vacutainer™ lithium heparin tubes without a gel for plasma separation, immediately before start of the surgery, and 2, 4, 6 and 24–36 h after the start of surgery. All surgeries were performed at Gävle Hospital. The blood samples were drawn either at surgery or in the postoperative ward. All blood samples were sent directly after blood sampling to the hospital laboratory, centrifuged within 2 h from venipuncture at 1890 *g* for 7 min. CRP and calprotectin were analyzed directly in freshly collected samples in Gävle Hospital. Remainder of each sample was frozen and sent to Uppsala University and analyzed later for IL-6, TNF-α, and procalcitonin. The study was performed in the period April 2019 till February 2020.

### Biomarker analysis

Calprotectin was measured in plasma samples with particle enhanced turbidimetric method (Gentian Diagnostics, Moss, Norway) on a Cobas c501 (Roche Diagnostics, Mannheim, Germany). Reagents, controls, and calibrators for calprotectin come from Gentian Diagnostics. Assay performances were checked using the manufacturer's control materials on two levels.

CRP was analyzed using the COBAS c501® system (Roche Diagnostics, Mannheim, Germany), using the CFAS-calibrator and Roche Modular® reagent. Assays were checked using the manufacturer's control materials on two levels. Procalcitonin, IL-6 and TNF-α were analyzed by sandwich ELISAs (DY8350, DY206, and DY210, R&D Systems, Minneapolis, MN, USA). The total coefficients of variation for the ELISA methods were approximately 6–7%. All assays were performed blinded without knowledge of clinical information.

### Statistics

All non-parametric statistics analyzes were performed using Analys-it for Excel and data displayed as mean ± 95% CI. Single-sample confidence interval estimation was performed using the formula: *μ* = *M* ± *t*(*sM*). *M* = sample mean, *t* = t statistic determined by confidence level, *sM* = standard error = √(*s*2/*n*). One-tailed t-test for 2 dependent means was used to calculate the difference between paired observations.

### Ethical considerations

The study was approved by the Uppsala Regional Ethics Committee, Uppsala, Sweden (Dnr 2018/227). All parts of the study were performed in accordance with the ethical approval and Swedish and European regulations. All participants gave written informed consent prior to inclusion in the study.

## Results

### Patient characteristics and early kinetics of calprotectin and CRP in plasma

Plasma samples were initially collected from 20 patients (18 men, age 41–79, 2 women, age 54–69), undergoing elective uncomplicated hernia repair with laparoscopic (*n* = 6) or open technique (*n* = 14). Patients could leave the study at any time point. Two of the initial 20 patients declined further participation in the study after the first preoperative sampling, and one patient was excluded from further participation because of hemolysis of pre-surgical blood sample, resulting in 17 patients to be finally included in the study (15 men and 2 women, pre-operative and at least one post-operative blood sample). Study samples for calprotectin and CRP were at 2 h obtained from 17 patients, at 4 h from 16 patients, at 6 h from 3 patients, and at 24–26 h from 9 patients. For analysis of IL-6, TNF-α, and procalcitonin samples from one patient were excluded because of sample freezing after shelf-life (24 h at room temperature). This resulted in study samples for IL-6, TNF-α, and procalcitonin being collected from 16 patients at 2 h, 15 patients at 4 h, 3 patients at 6 h, and 8 patients at 24–36 h.

**Figure 1.**
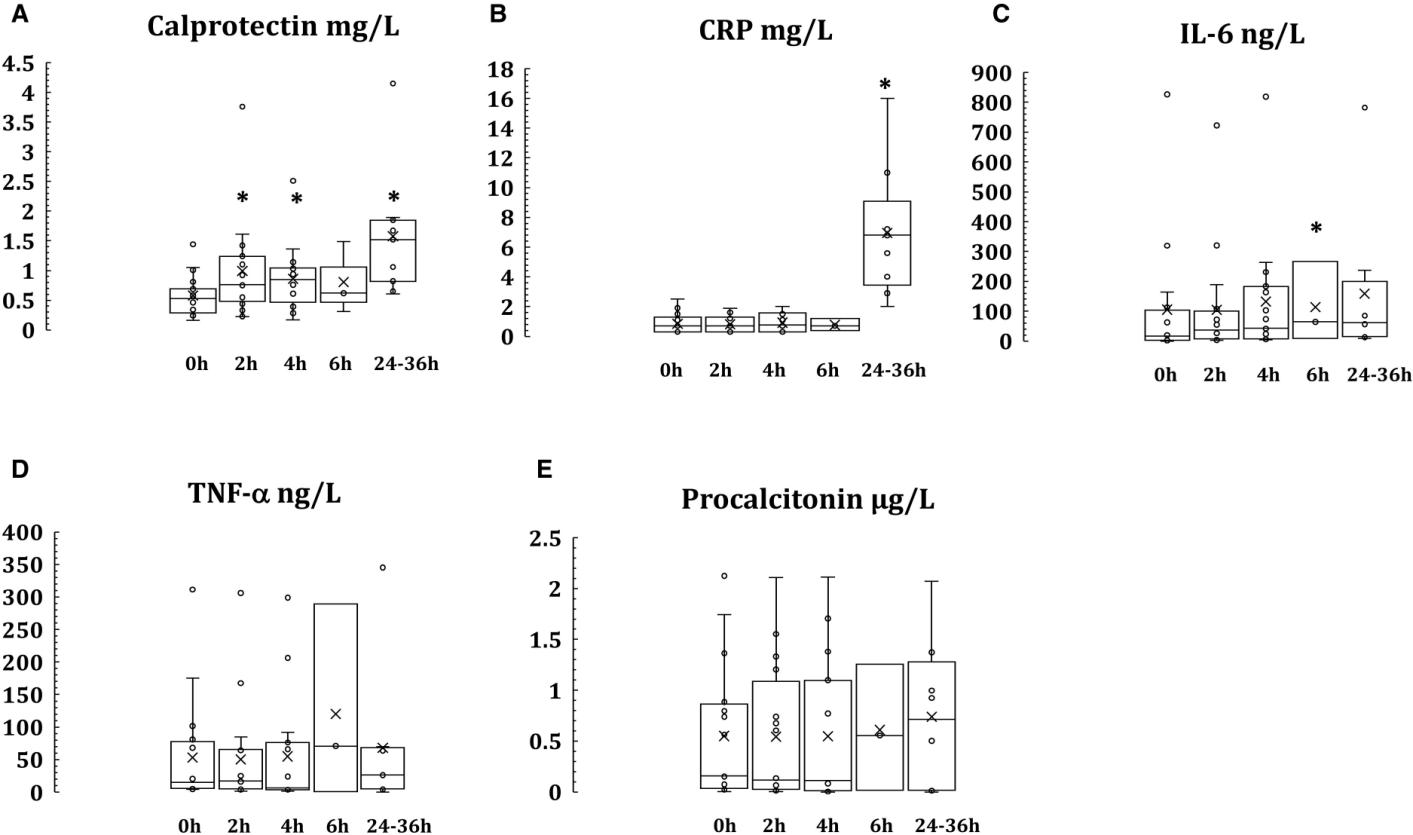
Early kinetics of calprotectin (A), CRP (B), IL-6 (C), TNF-α (D), and procalcitonin (E) in plasma following inguinal hernia surgery (before start of the surgery, and 2, 4, 6 and 24–36 h after the start of surgery). *Indicates statistical significance (*P* < 0.05).

Calprotectin levels in plasma were rapidly and significantly (*P* = 0.02532) elevated within 2 h following mild inflammatory response associated with inguinal hernia surgery, and continued to be elevated at all later time points analyzed, to reach the highest levels at the 24–36 h time point (Table 1 and Figure 1). IL-6 was found to be very transiently elevated 4 h post-surgery (*P* = 0.03098). CRP, on the other hand, remained within the normal reference levels 2, 4, and 6 h post-surgery, and was elevated (*P* = 0.00142) first at 24–36 h. No significant increase was observed at any analyzed time point post-surgery for TNF-α or procalcitonin (Tables 1 and 2 Figure 1).

**Table 1.** Calprotectin, CRP, IL-6, TNF-α and procalcitonin levels in plasma before start of the surgery, and 2, 4, 6 and 24–36 h after the start of surgery. Results of one-tailed t-test for 2 dependent means.

Analysis	Sample at time point (h)	Mean (M)	SD	*n*	Difference (Diff – M)	Sq. Dev	T	df	*P*
P-Calprotectin (mg/l)	0	0.58	0.34	17	0.41	10.42	2.11	16	0.02532*
	2	0.99	0.84						
	0	0.58	0.35	16	0.28	3.28	2.43	15	0.01410*
	4	0.86	0.56						
	0	0.56	0.26	9	1.02	7.50	3.15	8	0.00233*
	24–36	1.58	1.09						
P-CRP (mg/l)	0 h	0.85	0.67	17	−0.03	0.9	−0.51	16	0.30760
	2	0.82	0.57						
	0	0.88	0.67	16	0.04	1.72	0.44	15	0.33194
	4	0.92	0.62						
	0	0.98	0.57	9	5.98	143.12	4.24	8	0.00142*
	24–36	6.96	4.33						
P-IL-6 (ng/l)	0	105	210	16	−0.31	13,493.44	−0.04	15	0.48365
	2	105	185						
	0	108	217	15	24.13	29,719.73	2.03	14	0.03098*
	4	132	209						
	0	171	284	8	−12	8316	−0.98	7	0.17878
	24–36	159	262						
P-TNF-α (ng/l)	0	53	84	16	−2.81	836.44	−1.51	15	0.07635
	2	50	81						
	0	55	86	15	−0.27	1794.93	−0.09	14	0.46431
	4	55	87						
	0	65	104	8	2.88	1294.88	0.60	7	0.28437
	24–36	68	115						
P-Procalcitonin (ng/l)	0	0.55	0.68	16	−0.01	0.73	−0.11	15	0.45746
	2	0.54	0.67						
	0	0.55	0.70	15	0	0.06	0.11	14	0.45525
	4	0.55	0.72						
	0	0.72	0.74	8	0.02	0.2	0.27	7	0.39927
	24–36	0.74	0.75						

6 h, The sample size is too small to allow a reliable calculation of the t statistic.

*The results were considered significant at *P* < 0.05.

**Table 2.** Calprotectin, CRP, IL-6, TNF-α able procalcitonin mean ±95% CI values within [] in plasma before start of the surgery, and 2, 4, 6 and 24–36 h after the start of surgery.

Sample at timepoint (h)	Calprotectin (mg/l)	CRP (mg/l)	IL-6 (ng/l)	TNF-α (ng/l)	Procalcitonin (ng/l)
0	0.58, 95% CI [0.41, 0.75]	0.85, 95% CI [0.51, 1.20]	105, 95% CI [−6.9, 216.9]	53, 95% CI [8.24, 97.76]	0.55, 95% CI [0.19, 0.91]
2	0.99, 95% CI [0.56, 1.42]	0.82, 95% CI [0.53, 1.11]	105, 95% CI [6.42, 203.58]	50, 95% CI [6.84, 93.16]	0.54, 95% CI [0.18, 0.90]
4	0.86, 95% CI [0.56, 1.16]	0.92, 95% CI [0.59, 1.25]	132, 95% CI [16.26, 247.74]	55, 95% CI [6.82, 103.18]	0.55, 95% CI [0.15, 0.95]
6	0.81, 95% CI [−0.71, 2.33]	0.77, 95% CI [−0.22, 1.76]	113, 95% CI [−222.36, 448.36]	120, 95% CI [−252.62, 492.62]	0.61, 95% CI [−0.93, 2.15]
24–36	1.58, 95% CI [0.74, 2.42]	6.96, 95% CI [3.63, 10.29]	159, 95% CI [−60.04, 378.04]	68, 95% CI [−28.14, 164.14]	0.74, 95% CI [0.11, 1.37]

One patient had re-surgery because of postsurgical bleeding, but his calprotectin level was not higher than the reference interval (0–1.39 mg/l) at 24 h (0.61 mg/l), at a time point when CRP was slightly elevated 16 mg/l (reference interval < 5 mg/l).

## Discussion

The aim of the present study was to determine the early kinetics of calprotectin in plasma following a standardized temporary mild inflammatory response in comparison with other clinically used acute phase markers, using uncomplicated inguinal hernia surgery as a model. It is difficult to precisely define the onset of an infection with the precision required to be able to detect the inflammatory response within 2 h, and for this reason we chose to use a surgical model where we could precisely define the initial time of the inflammatory trauma. Calprotectin levels in plasma were rapidly and significantly elevated (within 2 h) following mild inflammatory response associated with inguinal hernia surgery, remained elevated at later time points, and reaching the highest level (1.58 mg/l) 24–36 h post-surgery. This contrasts to IL-6 and CRP, for which an elevation was found only later, with a transient elevation at 4 h post-surgery for IL-6, and an even later elevation for CRP in the 24–36 h post-surgery samples. Calprotectin also differs from response of TNF-α, and procalcitonin for which no significant increase at all was observed at any analyzed time point after surgery. This is in line with known kinetics of CRP following induction of inflammation/infection.^20,21^ The increase of calprotectin observed at least several hours prior to increase in CRP, as well as the stable elevation at least the first 24–36 h post induction of an inflammatory response may be of importance for early detection and assessment of inflammatory/infectious conditions. The rapid response of calprotectin may be due to the fact since calprotectin is stored in the neutrophils and can be released immediately without any *de novo* synthesis of the protein. Together with previous findings of calprotectin elevation in inflammatory and infectious conditions^6,13,15–17^ our results support the use of calprotectin as an early biomarker for inflammation/infection. Calprotectin concentration slowly increased during the first 2–36 h after surgery, demonstrating that calprotectin concentration did not exhibit short (< 24–36 h) peaks of elevation, indicating that there was no short “diagnostic window” for calprotectin to detect developing inflammation with a neutrophil activation. We speculate that a similar kinetics of calprotectin follow also more marked inflammatory responses seen in e.g., severe bacterial infections/sepsis. However, the infectious inflammatory response may differ from that elicited from the milder sterile inflammatory response studied here, necessitating further studies of early calprotectin kinetics also in infections. Furthermore, observations from this study indicated that calprotectin can be useful for detection of early postoperative infections since the levels were not significantly increased above normal reference range by the surgery itself. However, results should be interpreted with caution because of limitations of the study, including small sample size, unequal gender distribution, lack of coverage of younger age groups, the use here of only mild sterile inflammatory response, and lack of full series of blood samples. Obtaining a full series of blood samples from all the patients was hampered by the elective surgery patients being discharged early from the hospital at the time of stabilized condition, with subsequent samples requiring their active return to the hospital.

Further studies on calprotectin kinetics are required to establish the full kinetics of calprotectin following induction of inflammation/infection, to reveal the response to various sources of induction of inflammation/infection and to gain knowledge also of the later part of the kinetics. In conclusion, calprotectin levels in plasma were rapidly elevated (within 2 h following mild inflammatory response associated with inguinal hernia surgery and continued to increase at later time points (24–36 h)). Increase of calprotectin was observed several hours prior to increase in IL-6 or CRP. However, during the first 24 h the levels induced by the mild inflammatory response caused by inguinal hernia surgery were not significantly exceeding the normal reference range, suggesting that calprotectin can be useful for early detection of postsurgical infections.

## References

[1] World Health Assembly, 53. The world health report 2000: health systems: improving performance. Geneva: World Health Organization, 2000, https://apps.who.int/iris/handle/10665/79020 (accessed 2013/04/03)

[2] Dupuy AM Philippart F Péan Y , et al. Role of biomarkers in the management of antibiotic therapy: an expert panel review: I – currently available biomarkers for clinical use in acute infections. Ann Intensive Care 2013; 3: 22. 23837559 10.1186/2110-5820-3-22 PMC3708786

[3] Xu S Venge P . Lipocalins as biochemical markers of disease. Biochim Biophys Acta 2000; 1482: 298–307. 11058770 10.1016/s0167-4838(00)00163-1

[4] Havelka A Sejersen K Venge P , et al. Calprotectin, a new biomarker for diagnosis of acute respiratory infections. Sci Rep 2020; 10: 4208. 32144345 10.1038/s41598-020-61094-z PMC7060262

[5] Schenten V Plançon S Jung N , et al. Secretion of the phosphorylated form of S100A9 from neutrophils is essential for the proinflammatory functions of extracellular S100A8/A9. Front Immunol 2018; 9: 447. 29593718 10.3389/fimmu.2018.00447 PMC5859079

[6] Chan JK Roth J Oppenheim JJ , et al. Alarmins: awaiting a clinical response. J Clin Invest 2012; 122: 2711–2719. 22850880 10.1172/JCI62423 PMC3408740

[7] Vogl T Tenbrock K Ludwig S , et al. Mrp8 and Mrp14 are endogenous activators of toll-like receptor 4, promoting lethal, endotoxin-induced shock. Nat Med 2007; 13: 1042–1049. 17767165 10.1038/nm1638

[8] Ehrchen JM Sunderkötter C Foell D , et al. The endogenous toll-like receptor 4 agonist S100A8/S100A9 (calprotectin) as innate amplifier of infection, autoimmunity, and cancer. J Leukoc Biol 2009; 86: 557–566. 19451397 10.1189/jlb.1008647

[9] Donato R . S100: a multigenic family of calcium-modulated proteins of the EF-hand type with intracellular and extracellular functional roles. Int J Biochem Cell Biol 2001; 33: 637–668. 11390274 10.1016/s1357-2725(01)00046-2

[10] Berthier S Paclet MH Lerouge S , et al. Changing the conformation state of cytochrome b558 initiates NADPH oxidase activation: MRP8/MRP14 regulation. J Biol Chem 2003; 278: 25499–25508. 12719414 10.1074/jbc.M209755200

[11] Schenten V Bréchard S Plançon S , et al. iPLA2, a novel determinant in Ca2+- and phosphorylation-dependent S100A8/A9 regulated NOX2 activity. Biochim Biophys Acta 2010; 1803: 840–847. 20219570 10.1016/j.bbamcr.2010.02.006

[12] Choi IY Gerlag DM Herenius MJ , et al. MRP8/14 serum levels as a strong predictor of response to biological treatments in patients with rheumatoid arthritis. Ann Rheum Dis 2015; 74: 499–505. 24297376 10.1136/annrheumdis-2013-203923

[13] Menees SB Powell C Kurlander J , et al. A meta-analysis of the utility of C-reactive protein, erythrocyte sedimentation rate, fecal calprotectin, and fecal lactoferrin to exclude inflammatory bowel disease in adults with IBS. Am J Gastroenterol 2015; 110: 444–454. 25732419 10.1038/ajg.2015.6

[14] Foell D Roth J . Proinflammatory S100 proteins in arthritis and autoimmune disease. Arthritis Rheum 2004; 50: 3762–3771. 15593206 10.1002/art.20631

[15] Huang L Li J Han Y , et al. Serum calprotectin expression as a diagnostic marker for sepsis in postoperative intensive care unit patients. J Interferon Cytokine Res 2016; 36: 607–616. 27610929 10.1089/jir.2016.0037

[16] Simm M Söderberg E Larsson A , et al. Performance of plasma calprotectin as a biomarker of early sepsis: a pilot study. Biomark Med 2016; 10: 811–818. 27414210 10.2217/bmm-2016-0032

[17] Bartáková E Štefan M Stráníková A , et al. Calprotectin and calgranulin C serum levels in bacterial sepsis. Diagn Microbiol Infect Dis 2019; 93: 219–226. 30420210 10.1016/j.diagmicrobio.2018.10.006

[18] Jukić M Pogorelić Z Šupe-Domić D , et al. Comparison of inflammatory stress response between laparoscopic and open approach for pediatric inguinal hernia repair in children. Surg Endosc 2019; 33: 3243–3250. 30511312 10.1007/s00464-018-06611-y

[19] Sido B Teklote JR Hartel M , et al. Inflammatory response after abdominal surgery. Best Pract Res Clin Anaesthesiol 2004; 18: 439–454. 15212338 10.1016/j.bpa.2003.12.006

[20] Nakayama T Sonoda S Urano T , et al. Monitoring both serum amyloid protein A and C-reactive protein as inflammatory markers in infectious diseases. Clin Chem 1993; 39: 293–297. 8381732

[21] Barbić J Ivić D Alkhamis T , et al. Kinetics of changes in serum concentrations of procalcitonin, interleukin-6, and C- reactive protein after elective abdominal surgery. Can it be used to detect postoperative complications? Coll Antropol 2013; 37: 195–201. 23697273

